# Cyanobacteria and cyanophage contributions to carbon and nitrogen cycling in an oligotrophic oxygen-deficient zone

**DOI:** 10.1038/s41396-019-0452-6

**Published:** 2019-06-27

**Authors:** Clara A. Fuchsman, Hilary I. Palevsky, Brittany Widner, Megan Duffy, Michael C. G. Carlson, Jacquelyn A. Neibauer, Margaret R. Mulholland, Richard G. Keil, Allan H. Devol, Gabrielle Rocap

**Affiliations:** 1 School of Oceanography, University of Washington, Seattle, WA USA; 2 Horn Point Laboratory, University of Maryland, Cambridge, MD USA; 3 Geosciences Department, Wellesley College, Wellesley, MA USA; 4 Marine Chemistry and Geochemistry, Woods Hole Oceanographic Institution, Woods Hole, MA USA; 5 Department of Ocean, Earth and Atmospheric Sciences, Old Dominion University, Norfolk, VA USA; 6 Faculty of Biology, Technion—Israel Institute of Technology, Haifa, Israel

**Keywords:** Biogeochemistry, Microbial biooceanography

## Abstract

Up to half of marine N losses occur in oxygen-deficient zones (ODZs). Organic matter flux from productive surface waters is considered a primary control on N_2_ production. Here we investigate the offshore Eastern Tropical North Pacific (ETNP) where a secondary chlorophyll *a* maximum resides within the ODZ. Rates of primary production and carbon export from the mixed layer and productivity in the primary chlorophyll *a* maximum were consistent with oligotrophic waters. However, sediment trap carbon and nitrogen fluxes increased between 105 and 150 m, indicating organic matter production within the ODZ. Metagenomic and metaproteomic characterization indicated that the secondary chlorophyll *a* maximum was attributable to the cyanobacterium *Prochlorococcus*, and numerous photosynthesis and carbon fixation proteins were detected. The presence of chemoautotrophic ammonia-oxidizing archaea and the nitrite oxidizer *Nitrospina* and detection of nitrate oxidoreductase was consistent with cyanobacterial oxygen production within the ODZ. Cyanobacteria and cyanophage were also present on large (>30 μm) particles and in sediment trap material. Particle cyanophage-to-host ratio exceeded 50, suggesting that viruses help convert cyanobacteria into sinking organic matter. Nitrate reduction and anammox proteins were detected, congruent with previously reported N_2_ production. We suggest that autochthonous organic matter production within the ODZ contributes to N_2_ production in the offshore ETNP.

## Introduction

The oxygen content of the Pacific Ocean has been decreasing since the 1980s in response to increased oxygen utilization [1]. Model predictions suggest a 1% reduction in the Pacific’s O_2_ inventory will double the size of the Pacific oxygen-deficient zones (ODZs) [2]. Indeed, repeat measurements indicate that the Eastern Tropical North Pacific (ETNP) ODZ has thickened over the past 40 years and intruded into shallower waters [3]. In the absence of oxygen as an electron acceptor, oxidized nitrogen species can be reduced to N_2_ via two main pathways: heterotrophic denitrification and autotrophic anaerobic ammonia oxidation (anammox) [4, 5]. Both processes depend on organic matter, directly in the case of denitrification and indirectly in the case of anammox, which can use ammonium released during organic matter degradation as its reductant [6]. Organic matter flux has been correlated with N_2_ production in the environment [7–11], and indeed, the addition of sterilized sediment trap material significantly increased both denitrification and anammox rates in all three marine ODZs: the Arabian Sea, the Eastern Tropical South Pacific (ETSP), and the ETNP [5, 12]. Up to half of marine N losses from N_2_ production occur in marine ODZs [13], so an increase in the volume of ODZs may lead to an increase in N loss and stronger N limitation on primary producers globally.

Due to sluggish circulation [14], most of the volume of the ETNP ODZs is offshore, where productivity is 10× lower than in coastal regions [15]. In general, the phytoplankton communities responsible for organic matter production also differ between coastal and offshore regions, with larger eukaryotic phytoplankton more abundant in coastal regions while cyanobacteria are the dominant primary producers offshore [15]. Given this combination of low primary productivity rates and the lower export efficiency of communities dominated by smaller-celled phytoplankton [16], export fluxes are expected to be lower in the offshore ETNP. However, in addition to production in surface waters, populations of the cyanobacterium *Prochlorococcus* have been found at depth within all three major ODZs [17, 18] at a secondary chlorophyll *a* maximum where the 1% of the deeply penetrating blue irradiance (490 nm) overlaps with the ODZ [19]. Genetic evidence using the 16S–23S ribosomal RNA (rRNA) internal transcribed spacer (ITS) region suggests that this population may be composed of novel lineages of *Prochlorococcus* that are closely related to cultured isolates of the low light IV (LLIV) ecotype [20]. At five stations in the ETNP, primary production was measured at the *Prochlorococcus*-dominated secondary chlorophyll *a* maximum [21]. It has been suggested that in situ production in the ODZ could significantly increase the organic C supply to N_2_-producing heterotrophs [21]. Here we show productivity and export measurements along with metaproteomic and metagenomic analyses that strongly supports this hypothesis. The pathway to deliver organic carbon fixed by *Prochlorococcus* cells to heterotrophic denitrifiers has not been established in the ODZ. Viruses have been implicated in organic matter cycling in the ocean [22], and abundance of cyanobacterial viruses have been shown to strongly correlate with organic matter flux in the oxic oligotrophic ocean [23]. From the abundance and composition of cyanobacterial viruses on particles, we suggest a role for these viruses in the conversion of cells into sinking organic matter in the ODZ.

## Methods

### Sampling

Samples were collected in April 2012 aboard the *R/V Thompson* during cruise TN278 using 10 L Niskin bottles attached to a 24 bottle CTD (conductivity–temperature–depth) rosette. A Seabird 911 Conductivity Temperature Density meter, a Seabird SBE 43 Dissolved Oxygen Sensor, a WETLabs ECO Chlorophyll Fluorometer, and a Biospherical/Licor PAR/Irradiance Sensor were attached to the CTD rosette. Hydrographic and nutrient data from the cruise are deposited at http://data.nodc.noaa.gov/accession/0109846.

Three free-floating surface-tethered sediment net traps [24] were deployed at station BB2 at 105, 150, and 750 m. Sediment trap carbon and nitrogen fluxes were measured as in Babbin et al [5]. Logistics precluded duplicate measurements of sediment trap-derived flux estimates. Carbon flux from 105 m was previously published [5], but carbon fluxes from 150 and 750 m and nitrogen fluxes have not been previously published.

### Productivity measurements

**Fig. 1:**
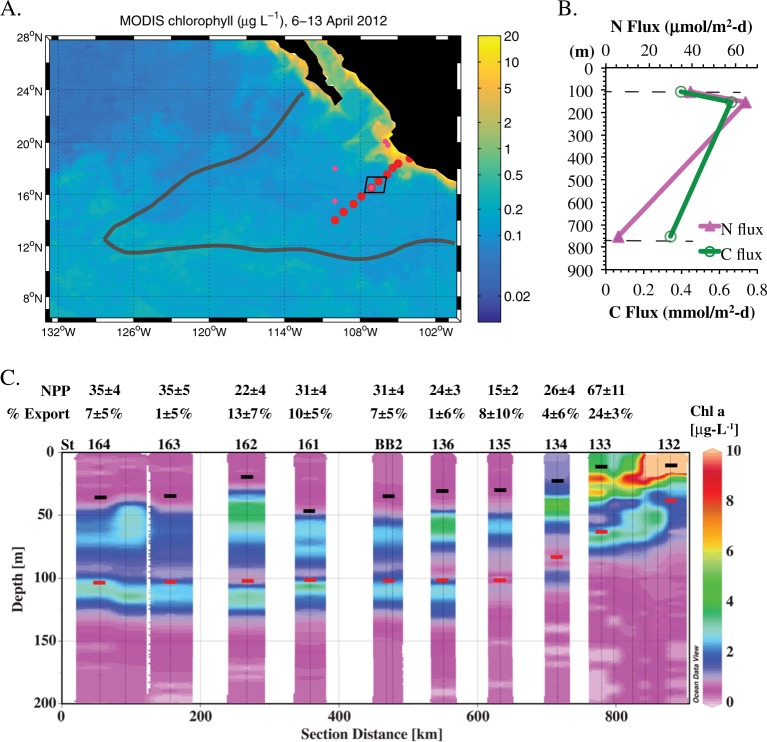
**a** Map of 8-day averaged satellite chlorophyll over the time period of sampling. Red dots indicate productivity sampling stations where mixed layer triple oxygen isotopes and oxygen/argon dissolved gas ratios were measured and small pink dots indicate stations where ^13^C incubations took place. Dark gray line indicates 10 µM O_2_ oxygen-deficient zones (ODZ) boundary as determined from World Ocean Atlas. A box indicates stations 136 and BB2. **b** Sediment trap data from station BB2. Dashed lines indicate ODZ boundary. **c** Transect of chlorophyll *a*. Station numbers are listed above the graph and correspond to triple oxygen isotope (TOI) productivity measurements (NPP; mmol C m^−2^ day^−1^) and export efficiency. Black dashes indicate mixed layer depth, and red dashes indicate the top of the ODZ

Samples for triple oxygen isotope (^17^Δ; TOI) and O_2_/Ar dissolved gas analysis were collected in duplicate from the surface mixed layer on a coastal to open ocean transect (station locations shown in Fig. 1 and station information listed in Table S1) and analyzed following the laboratory procedures described in Palevsky et al. [25]. Gross primary production (GPP) rates can be calculated from mixed layer TOI by leveraging the unique isotopic signature of atmospheric-derived oxygen, which is depleted in ^17^O as compared to photosynthetically derived oxygen due to mass-independent fractionation in a stratospheric photochemical reaction. The TOI system is insensitive to mass-dependent fractionation as respiration removes oxygen, isolating the influence of photosynthesis from respiration and enabling calculation of mixed layer GPP by combining TOI measurements with air–sea gas exchange rates [26]. Net community production (NCP) rates, which represent organic carbon export from the mixed layer under steady-state conditions, can similarly be calculated by combining biological oxygen supersaturation determined from measured O_2_/Ar with air–sea gas exchange rates.

Mixed layer GPP and NCP rates were calculated from estimated air–sea gas transfer velocity and measured TOIs ([27]; Eq. 7) and O_2_/Ar ([28]; Eq. 3), respectively. These equations assume steady-state conditions and negligible influence of mixing and advection in the mixed layer dissolved gas budget, which model results show to be a reasonable approximation for the ETNP in April [29]. Daily wind speed data from National Oceanic and Atmospheric Administration National Climatic Data Center’s multiple-satellite Blended Sea Winds product (https://www.ncdc.noaa.gov/oa/rsad/air-sea/seawinds.html) were used to calculate air–sea gas transfer velocity [30] with the Reuer et al. [28] weighting scheme. Uncertainty in GPP and NCP rates were determined using a Monte Carlo approach (as in [25]). Mixed layer GPP rates were converted to rates equivalent to incubation-based net primary production (NPP) using a gross O_2_:net C ratio of 2.7 [31]. NCP rates were converted to carbon units using a O_2_:C ratio of 1.1 appropriate for production using a recycled nitrogen source [32].

Volumetric NPP was measured on 13 CTD casts at 10 stations (multiple casts at stations BB1 and BB2; Table S2) using stable isotope highly labeled (^13^C; 99%) bicarbonate in 24 h incubations. Whole water was transferred directly from Niskin bottles to 1 L polyethylene terephthalate glycol bottles using inert tubing. Incubations in triplicate light and duplicate dark bottles were initiated before dawn with the addition of a 10% enrichment of ^13^C-sodium bicarbonate, and bottles were incubated in shipboard incubators equipped with flow thru surface seawater to maintain temperature and neutral density screens to simulate in situ light levels where samples were collected. After 24 h, incubations were terminated by filtration (≤127 mmHg) of the entire bottle onto a pre-combusted (2 h at 450 °C) GF/F filter, and filters were stored at −20 °C. Upon return to the lab, filters were dried at 40 °C, pelletized in tin capsules, and analyzed on a Europa 20/20 isotope ratio mass spectrometer equipped with an automated nitrogen and carbon analyzer. Independent measurements of the initial particular carbon concentration and particle isotopic enrichment were used to calculate net primary production. Rates of carbon fixation were calculated as in Mulholland and Capone [33], and rates in the dark were subtracted from rates measured in light incubations.

Satellite chlorophyll data for 6–13 April 2012 (MODIS Aqua R2014, available from http://www.science.oregonstate.edu/ocean.productivity) was extracted from a 1/3° box around each station.

### Metagenomics

Metagenomic libraries constructed, sequenced, and assembled using 125 and 150 bp paired end sequences at station 136 (−106.543°W 17.043°N) and station BB2 (107.148°W 16.527°N; cast 141) were previously published in Fuchsman et al [34]. DNA samples for the depth profile at station 136 were filtered onto a 0.2 μm SUPOR filters. At station BB2, DNA samples were filtered through 30 μm filters at 100, 120, and 150 m depths and subsequently filtered onto a 0.2 μm SUPOR filter. At 120 m, this prefiltered sample (30–0.2 μm fraction) was also sequenced. Depth and pore size of filters used for metagenomic analysis are found in Table S1. Hydrographic conditions were very similar at these two stations that were only 83 km apart [34]. Metagenomic sequences were assembled independently from each sample into larger contigs using the VELVET (1.2.10) assembler [35], as described in Fuchsman et al. [34] and the MEGAHIT assembler [36] as described in Widner et al [37]. Assembled contigs were functional annotation by PROKKA [38]. ETNP 2012 metagenomic reads and assembled contigs can be found at GenBank bioproject PRJNA350692.

Although the metagenomic sequences have been previously published, the genes of interest for this paper (*psbD* and DNA polymerase A and B) have not been previously examined. As in Fuchsman et al. [34], for each gene, a reference phylogenetic tree was constructed using both published full-length and full or nearly full-length sequences assembled from the metagenomes themselves. Sequences of interest from the metagenomes were found by searching a custom blast database [39] of all our assembled open reading frames as called by Prodigal [40] and annotated with Prokka [38], using representative published sequences as query sequences. All sequences recruited from blast were combined with the previously published full-length gene sequences and aligned in amino acid space with MUSCLE v. 3.8.1551 [41]. Maximum likelihood phylogenetic trees were constructed with the reference sequence alignments of the genes of interest using the program RAxML v. 8.1.20 [42]. The trees were constructed with a gamma model of rate heterogeneity, and appropriate amino acid substitution models were determined for each tree. Bootstrap analyses (*n* = 100) were performed on each tree. For the rRNA ITS tree a replicate of the Lavin et al. [20] phylogenetic tree was used.

A phylogenetic placement approach [43] was used to characterize short metagenomic reads related to the targeted genes of interest (psbD, viral DNA polymerase, and the ITS region of rRNA), in a semi-quantitative and phylogenetically specific manner [34, 44]. This allows us to both confirm the accuracy of a read assignment to a gene and also to differentiate between different taxa that might have been sources for the read. For read placement, the short metagenomic reads were recruited via tblastn search with an *e* value cutoff of <−5 [39]. The recruited reads were trimmed to the edge of the gene of interest to remove any overhang of up- or down-stream sequence, trimmed to the proper reading frame of the blast results. DNA polymerase and psbD reads were converted to amino acid space, while ITS reads remained in nucleotide space. Any sequence ambiguities and stop codons were removed. Only sequences longer than 100 bp (33 amino acids) after quality trimming were used. These reads were aligned to the reference sequences using PaPaRa: Parsimony-based Phylogeny-Aware Read Alignment program [45]. Paired end reads were combined into the same alignment and placed as one on the tree using the EPA: Evolutionary Placement Algorithm portion of RAxML [42]. Each read has a number, or “branchlength,” which corresponds to the similarity between the read and the sequence to which it is placed. Reads placed with a read “branchlength” longer than 2.0 were removed as erroneous. Spot testing indicated that these reads belonged to different genes than the one examined. Only a small percentage of reads were thus removed (0.1–2.5%). Reads are normalized by the number of sequences in each metagenome, divided by gene length, and then multiplied by 100 to make the numbers easier to visualize. For the ITS region, read abundance for *Synechococcus*, *Prochlorococcus* LLIV/V, and NC1 were divided by two since those organisms contain two copies of the ITS region.

### Metaproteomics

At station BB2 metaproteomic analyses were conducted on both water column and sediment trap samples. Water was filtered in situ at 55, 100, 145, 160, and 250 m using a McLane pump (McLane Research Laboratories, Falmouth, MA, USA) fitted with a 4 mm mesh screen and combusted 142 mm diameter GF/F filters (0.7 µm) that were double-stacked. Volumes filtered were between 300 and 1000 L. Depth and filter pore size used for metaproteomic analysis are found in Table S3. Materials from two 11 mm circular punches of the GF/F filter were used for protein extraction. Additionally proteins were extracted from two 0.2 µm Sterivex filters from 100 and 150 m (~6 L of water filtered from the CTD) to compare to the results from the larger GF/F pore size from the McLane pumps. The sediment trap samples were allowed to settle and then decanted to a small volume and split between carbon and proteomics analyses. Only 105 and 750 m sediment trap samples were analyzed for proteomics. Filters or sediment trap material were extracted into ammonium bicarbonate following a protocol adapted from Bridoux et al [46]. Briefly, the chilled suspension was lysed with the help of a high-power sonicator, resulting in a whole-debris lysate. The lysate was enzymatically digested with trypsin, following a protocol adapted from Nunn et al. [47]. The resulting tryptic peptides were desalted using a macro-spin C18 column (NestGroup), resuspended in 5% acetonitrile with 0.1% formic acid (FA), and 1 µg of protein was introduced to a Thermo Q Exactive Plus mass spectrometer using a Waters nanoAcquity UPLC. Peptides were separated on a home-packed analytical column consisting of a 37 cm log, 75-μm i.d. fused-silica capillary column packed with C18 particles (Magic C18AQ, 100 Å, 5 mm; Michrom) coupled to a 4 cm long, 100 μm i.d. precolumn (Magic C18AQ, 200 Å, 5 mm; Michrom). Solvents of 100% water with 0.1% FA (A) and 100% acetonitrile with 0.1% FA (B) were used to elute peptides over a 90 min gradient from 5 to 35% solvent B. A data-dependent acquisition strategy was utilized, where tandem mass spectrometry spectra were collected on the ten most intense ions observed in the MS^1^ precursor scan.

The resulting mass spectra were analyzed using PEAKS (version 8.5; Bioinformatics Solutions, Waterloo, Canada; [48]). Proteins were identified using PEAKS DB, a database matching search platform augmented by de novo peptide sequencing, using a database consisting of assembled proteins from ETNP metagenomes [34, 49, 50] as well as all proteins translated from the Marine Microbial Eukaryote Transcript Project [51] and a selection of genomes from relevant cultures (Table S4). A contaminant database downloaded from http://www.thegpm.org/crap/ was included in the database search. A target-decoy search approach was used to estimate the false discovery rate of peptide-spectrum matches. All MS^2^ spectra were searched against the database with the following parameters: enzyme type, trypsin; allowed missed cleavages, 3; peptide confidence cutoff: ≥15 −log *P*; protein confidence cutoff: ≥20 –log *P*; unique peptides: ≥1; protein false discovery rate: 4%. Fixed modification of cysteine residues by 57.021 Da, due to iodoacetamide modification, and variable modification of methionine by 15.995 Da, due to oxidation, were allowed. Protein identifications were accepted if they could be established at >95.0% probability.

When the identified protein was a protein from assembled ETNP metagenomes, in an attempt to identify the organism the protein derived from, the protein was blasted against a custom database composed of the proteins from genomes used for the proteomics database (Table S4). The best blast hit was recorded if the *e* value was <−20.

The phylogenetic composition of the proteins identified from the GF/F and 0.2 μm Sterivex filters from the same depths are fundamentally similar with some variations in the abundance of taxa (Fig. S3). The percent of cyanobacteria decrease and the amount of Marine Group A/SAR406 increase in 0.2 μm samples compared to GF/F (Fig. S3).

## Results and discussion

### Productivity and export

**Fig. 2:**
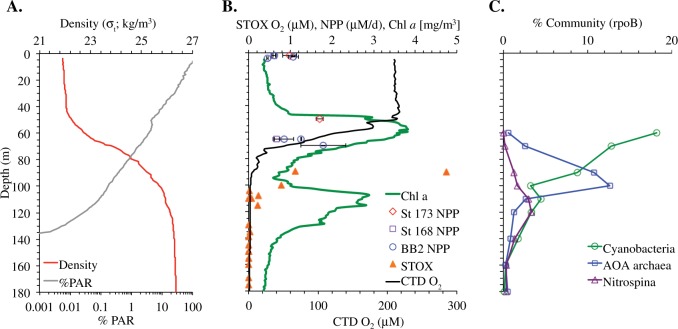
Primary production, autotrophic bacteria, and water column properties offshore. **a** %PAR (photosynthetically active radiation) and density profiles at station BB2, **b** Chlorophyll *a* fluorescence, STOX (Switchable Trace Oxygen) oxygen data from repeat casts at BB2 [54], net primary productivity (NPP) from ^13^C experiments from three offshore stations. **c** Metagenomic *rpoB* reads for cyanobacteria, and oxygen-utilizing ammonia-oxidizing Thaumarchaea (AOA) and nitrite oxidizer *Nitrospina* at station 136 [34]

In April 2012, we occupied 10 stations along an 830 km transect from the coast (18.8°N 104°W) to offshore waters (14.0°N 110°W) through the heart of the ETNP ODZ (Fig. 1a). We use the triple oxygen isotope composition of mixed layer dissolved oxygen to calculate mixed layer productivity across the spatial extent of the transect and ^13^C enrichment incubations to expand our productivity measurements below the mixed layer at a few stations. Satellite-derived surface chlorophyll *a* concentrations were 20 μg L^−1^ at the station nearest the coast, decreasing greatly to 0.1–0.3 μg L^−1^ at offshore stations (Fig. 1, Table S1). These offshore chlorophyll *a* values are typical of the region [15]. Similarly, gross primary production in the mixed layer, determined by the incubation-independent triple oxygen isotope (TOI) technique, was highest near the coast (St 133: 182 ± 29 mmol O_2_ m^−2^ d^−1^) and lower (41 ± 7 to 94 ± 12 mmol O_2_ m^−2^ d^−1^) at the more offshore stations (Table S1). Using a gross O_2_:net C ratio of 2.7 [31], these values are equivalent to net primary production (NPP) offshore of 15 ± 2 to 34 ± 5 mmol C m^−2^ d^−1^; Table S1 and Fig. 1). Volumetric NPP rates measured using ^13^C incubations were approximately ten times greater in surface waters at the coastal stations (5–11 µM C d^−1^ or 71–130 mmol C m^−2^ d^−1^ integrated across the mixed layer; Figure S1 & Table S2) than in the offshore region (0.5–1 µM C d^−1^ or 16–29 mmol C m^−2^ d^−1^ integrated across the mixed layer) (Fig. 2 & Table S2). In the literature, the average surface productivity in this offshore region is 0.2 µM C d^−1^ [15], even smaller than our measured surface values. TOI and ^13^C NPP estimates agree well given that TOI measurements represent an average over the mixed layer over several weeks while the incubation experiments were 24 h.

The majority of the offshore stations exhibited vertical profiles with two chlorophyll *a* maxima; both were below the surface mixed layer and the secondary chlorophyll *a* maximum was within in the upper ODZ (Fig. 1c). Mixed layer TOI samples only provide estimates of productivity within the mixed layer, but ^13^C incubations were conducted from the surface to 1% PAR at five stations to determine sub-mixed layer primary production rates (Table S2). At the offshore stations where ^13^C-NPP incubations were conducted, volumetric rates at the primary chlorophyll *a* maximum varied between 0.7 and 1.8 µM C day^−1^ (Fig. 2; Table S2). Thus, at the offshore stations volumetric NPP rates were similar from the surface to 1% PAR (Fig. 2). At station BB2, volumetric NPP integrated from the mixed layer down to the 1% PAR level at 75 m (Fig. 2) for ^13^C-based NPP measurements was 49 mmol C m^−2^ day^−1^, only slightly higher than the mean euphotic zone NPP at oligotrophic Station ALOHA (42 ± 7 mmol C m^−2^ day^−1^) [52].

At the offshore stations, carbon export from the mixed layer, as determined from dissolved oxygen/argon ratios, ranged from 0.2 to 3.2 mmol C m^−2^ day^−1^, or 1–13% of NPP (Table S1 and Fig. 1). Thus, the majority of photosynthetically fixed carbon was recycled within the mixed layer, with only a small fraction sinking out of the surface mixed layer. These export rates are lower than mean export from the mixed layer determined by dissolved oxygen/argon ratios at Station ALOHA near Hawaii (10 ± 3 mmol C m^−2^ day^−1^; [52]). Export rates were also lower than offshore stations off the coast of Southern California (9 ± 3 mmol C m^−2^ day^−1^) where mixed layer net productivity from TOI was 31 ± 14 mmol C m^−2^ day^−1^ [53]. However, the rates reported here in the ETNP do not include contributions to export from below the mixed layer, which need to be considered since the mixed layer was shallower than the euphotic depth throughout the study region and both chlorophyll *a* maxima were substantially below the surface mixed layer. Sediment trap flux estimates from traps deployed at 105 m at station BB2 (the top of the ODZ as determined by a STOX (Switchable Trace Oxygen) oxygen sensor; [54]) were lower (0.39 mmol C m^2^ day^−1^ [5] and 38 μmol N m^2^ day^−1^; Fig. 1b) than those estimated at the bottom of the mixed later (42 m; Fig. 2) by the TOI method (Table S1), indicating that carbon export 60 m below the mixed layer was also low. These flux rates were lower than sediment trap-based flux estimates at a similar depth from the persistently oligotrophic regime at Station ALOHA (1.7–3.3 mmol C m^−2^ day^−1^; [55]).

Although organic matter fluxes generally attenuate with depth in the ocean, at our station in 2012, sediment trap fluxes at 150 m (0.66 mmol C m^−2^ day^−1^ and 64 μmol N m^2^ day^−1^) were higher than fluxes measured at 105 m (Fig. 1b). The 750 m sediment trap did measure the lowest fluxes (0.34 mmol C m^−2^ day^−1^ and 6 μmol N m^−2^ day^−1^), however. If we assume the difference between fluxes at 105 and 150 m (0.26 mmol C m^−2^ day^−1^ and 25 μmol N m^−2^ day^−1^) are due to in situ productivity in the ODZ, this productivity would make up 40% of the particle flux at 150 m. Although no primary production measurements were made within the secondary chlorophyll *a* maximum (110–125 m) in the ODZ during the 2012 cruise, rates of NCP integrated across the secondary chlorophyll *a* maximum of 0.13– 0.93 mmol C m^−2^ day^−1^ were measured by ^13^C incubations at five stations in a similar area of the ETNP in 2014 [21], demonstrating the potential for in situ productivity within the ODZ. Although our calculation of a 40% contribution to flux involves a comparison of only two sediment trap samples, combined with previously measured productivity rates in the ODZ, these data suggest a significant amount of primary production and carbon export at the secondary chlorophyll *a* maximum.

### Phytoplankton in and above the ODZ

To characterize the functional and taxonomic diversity of microbes within this oligotrophic ODZ community, we examined metagenomes from this 2012 cruise [34] from nine depths (60–180 m) at the offshore station 136 (Fig. 1a) with particular attention to the secondary chlorophyll *a* maximum. We identified short sequences by both function and phylotype by placing them on reference trees constructed from full-length genes, allowing us to use the short-read data in a semi-quantitative manner.

**Fig. 3:**
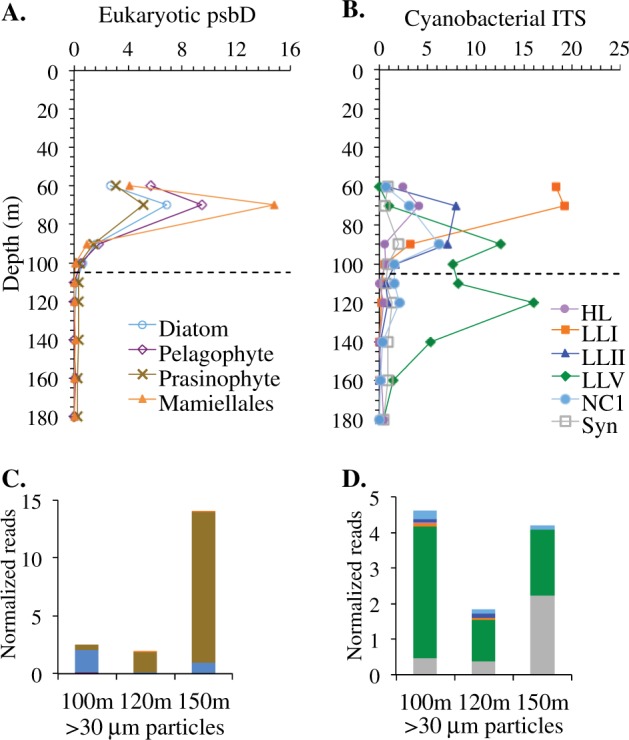
Diversity of photoautotrophs in the water column and on particles. **a** Depth profile of normalized reads for eukaryotes as determined by *psbD* in water column metagenomes. **b** Depth profile of normalized reads for cyanobacterial genotypes based on internal transcribed spacer (ITS) sequences in water column metagenomes, including *Synechococcus* (Syn) and *Prochlorococcus* high light (HL) and low light (LL) clades and the uncultured NC1 clade. **c** Eukaryotic *psbD* normalized reads found in 100, 120, and 150 m particle metagenomes. Colors indicate *psbD* phylotypes as indicated in **a**. **d** Cyanobacterial ITS genotypes from normalized reads found in 100, 120, and 150 m particle metagenomes. Colors indicate ITS genotypes as indicated in **b**. Dashed lines indicate the top of the oxygen-deficient zones (ODZ)

We used genes for the photosystem I reaction center core protein PsbD to identify photoautotrophs (Fig. S2). The primary chlorophyll *a* maximum contained both cyanobacteria and eukaryotes, including green algae (an unidentified prasinophyte and Mamiellales related to *Ostreococcus* and *Bathycoccus*) and heterokonts (Pelagophytes and diatoms) (Fig. 3a, b). In contrast, the secondary chlorophyll *a* maximum was entirely cyanobacteria with *Prochlorococcus* dominating the photoautotrophic community (Fig. 3a, b), which is consistent with previous flow cytometry results [17, 21]. The proportion of cyanobacterial reads declined with depth from 18% of the total microbial community in oxic waters, as determined by rpoB, to about 5% in the upper ODZ (Fig. 2c). We used the rRNA ITS to further identify the *Prochlorococcus* genotypes in each region of the water column. *Prochlorococcus* clade LLI was dominant at 60 m (Fig. 3), which corresponds to the primary chlorophyll *a* maximum (Fig. 2), and *Prochlorococcus* clades LLII and the uncultured clade NC1 [56] were also found in oxic waters (Fig. 3b). *Prochlorococcus* clade LLIV/LLV was the dominant ecotype in the anoxic secondary chlorophyll *a* maximum (Fig. 3b).

Additionally, we performed metaproteomics on water column samples (McClane pump) at five depths at station BB2 (55, 100, 145, 160, and 250 m), Cyanobacteria dominated the metaproteome at 100 m composing 50% of the total identified proteins (Fig. S3; Table S5). The number of cyanobacterial proteins decreased with depth, composing 22% of identified proteins at 145 m and 10% at 160 m, and 4% at 250 m (Fig. S3; Table S5). *Prochlorococcus*-derived proteins exceeded those from *Synechococcus* in the water column (Fig. S3; Table S5), probably due to the higher abundance of *Prochlorococcus* in the community (Fig. 3). Although our analysis was not quantitative and many proteins present in the sample were likely not identified due to the database-dependent limitations of the metaproteomics technique [57], the presence of a protein in metaproteomic sample, particularly one that might be expected to have a short half life, such as an enzyme, suggests it is being actively expressed.

**Fig. 4:**
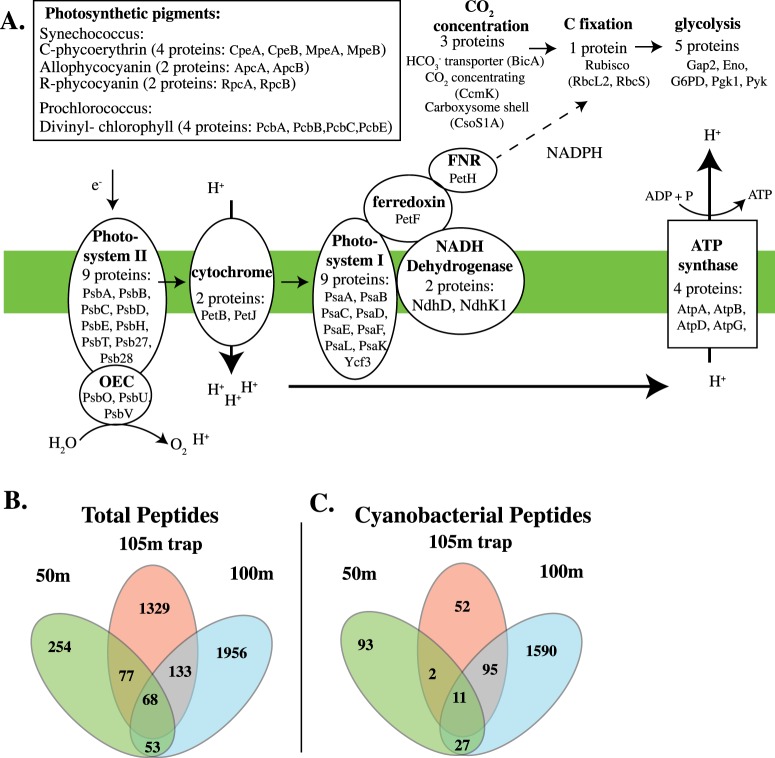
Metaproteomic data indicating active cyanobacterial production within the oxygen-deficient zones (ODZ) and evidence for the ODZ cyanobacterial population contribution to particles. **a** Cyanobacterial proteins involved in primary production identified at 100 m. The Oxygen evolving complex (OEC) and ferredoxin NADP+ reductase (FNR) are abbreviated on the figure. **b** A comparison of total peptides identified between the 105 m sediment trap, 50 m oxic water column, and 100 m ODZ water column samples. **c** A comparison of only the cyanobacterial peptides identified between the 105 m sediment trap, 50 m oxic water column, and 100 m ODZ water column samples

Identified proteins indicate that cyanobacteria were actively photosynthesizing and fixing carbon in the water column at 100 m, a depth with 0.8 μM oxygen [54]. Proteins identified included those used in light capture, cyanobacterial enzymes for photosynthesis (28 photosystem proteins, including all 3 extrinsic proteins of the oxygen evolving complex), electron transport (cytochrome *c*6 and ferredoxin), ATP synthesis (4 subunits of ATP synthase), bicarbonate transporters, carbon concentration (carboxysome shell protein and CO_2_ concentrating protein CcmK), and carbon fixation (both subunits of RubisCo), as well as replication (DNA-binding protein HU), transcription (2 subunits of DNA directed RNA polymerase), translation (Elongation Factors Tu, Ts, and G), and cell division (FtsZ) were all detected (Fig. 4a, Table S5). Additionally, *Prochlorococcus* were actively attempting to acquire nitrogen compounds for assimilation as evidenced by detection of nitrogen regulatory protein P-II, a urea ABC transporter, a nitrite transporter, and an assimilatory nitrite reductase. Since ammonium in the ODZ is generally below detection limits [37], *Prochlorococcus* populations may need to use alternative sources of N for assimilation. Thus, *Prochlorococcus* was clearly active at the secondary chlorophyll *a* maximum in the ODZ.

### Cyanobacteria on particles

To investigate whether ODZ cyanobacteria can contribute to the particles that make up the sinking organic matter in the ODZ, we examined metagenomes from the >30 μm size fraction collected from Niskin bottles at 100, 120, and 150 m, and metaproteomes from sediment trap material at 105 and 750 m, both at offshore station BB2. Cyanobacteria were present in >30 μm particles, especially at 100 m, where, when normalized by the single copy core gene *rpoB*, they made up 7.5% of the prokaryotic community [34]. In contrast, we note that the % of community of SAR11, the most abundant bacteria in the ODZ, decreased dramatically on the >30 μm particles compared to the water column [34], indicating that the 30 μm filters were not clogged. The cyanobacteria in the particle fraction were predominately LLIV/LLV *Prochlorococcus*, prevalent in the water column in the ODZ, and *Synechococcus* group CRD1, which was present in the oxic water column but not within the ODZ water column (Fig. 3d). The difference in *Synechococcus* types between particles and the ODZ water column indicates that *Synechococcus* on particles came from surface waters. *Synechococcus* has been found on particles in other systems [58]. Notably, the fraction of LLI *Prochlorococcus*, dominant at the primary chlorophyll *a* maximum, was miniscule at the secondary chlorophyll *a* maximum and in the particle fraction (Fig. 3d), implying that in contrast to *Synechococcus, Prochlorococcus* cells were not sinking into the ODZ from the primary chlorophyll *a* maximum.

In the 105 m sediment trap metaproteomes, cyanobacterial proteins composed 17% of the identified proteins (Fig. S3). The cyanobacterial proteins consisted of structural proteins used in light capture, proteins used in photosynthesis, ATP synthesis, and transporters (Table S5). When cyanobacterial peptides were compared between the oxic 50 m water column sample, the 100 m ODZ water column sample and the 105 m sediment trap, 67% of the cyanobacterial peptides in the 105 m trap were shared with the 100 m water column sample (Fig. 4c). Thus, our particulate metaproteomic data and metagenomic data both suggest that there is transfer of some organic material from cyanobacteria living in the ODZ to sinking particles.

### Viruses as source of cyanobacterial mortality

**Fig. 5:**
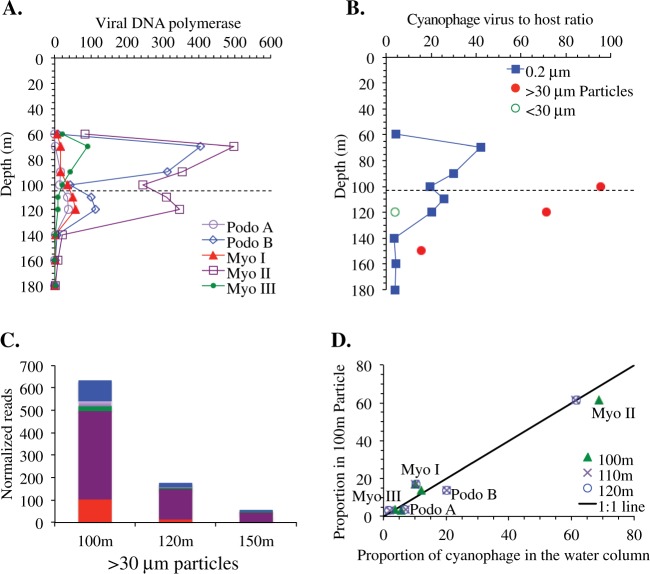
Cyanophage prevalence in the water column and on particles. **a** Depth profiles of normalized reads for DNA polymerase phylotypes. **b** Virus to host ratio in cells (>0.2 μm) and on particles (>30 μm) and one free-living microbial sample (<30 μm). Ratios are calculated using cyanophage DNA polymerase divided by cyanobacteria RNA polymerase B (*rpoB*). Dashed line indicates the top of the ODZ. **c** Viral DNA polymerase normalized reads found in 100, 120, and 150 m particle metagenomes. Colors indicate DNA polymerase phylotypes as indicated in **a** and delineated in Figs. S4, S5. **d** Comparison of the proportion of DNA polymerase phylotypes between 100 m particulate (>30 μm) and >0.2 μm water column samples from the ODZ (100, 110, and 120 m). Each symbol represents the proportion of a phylotype of DNA polymerase. The black line represents the 1:1 line

We evaluated cyanophages (viruses that infect cyanobacteria) as a potential contributor to cyanobacteria mortality and particle formation. Cyanophages were detected in both free-living and particle metagenomes using DNA polymerase A and B, forms of DNA polymerase found in all known T7-like cyanopodoviruses and T4-like cyanomyoviruses (Fig. 5). Genes for cyanophage DNA polymerase can be split into phylotypes (Fig. 5 and Figs. S4, S5; following [59]). Cyanomyoviruses were more abundant than cyanopodoviruses in the ODZ with cyanomyovirus clade II as the dominant type of virus (Fig. 5A). While cyanomyovirus clade III decreases in the ODZ, cyanomyovirus clade I and cyanopodovirus A increase in the ODZ (Fig. 5A). Thus, the relative proportions of cyanophage types differ between the oxic waters and the ODZ. The virus/host ratio (as calculated using cyanophage DNA polymerase/cyanobacteria *rpoB*) was high (30–40) in the free-living metagenomes and even higher (>100) in the >30 μm particulate metagenomes, which is consistent with active infection (Fig. 5B). Viral infection has been shown to cause bacteria to clump and form particles [60]. Although sinking particles may also scavenge viruses from the water column [61], the cyanophage phylotypes on 100 and 120 m particles are found in the same proportions as found in the ODZ water column, but differed in proportion compared to those in the oxic overlying water (Fig. 5C, D and Fig. S6). This is consistent with the idea that cyanobacteria and their viruses were not sinking into the ODZ from particles formed in oxic water above, but rather were creating particles in situ.

Viral proteins were also detected in the metaproteomes from the water column and the sediment trap. At 100 m many structural capsid proteins for cyanophage were identified in the proteome (Table S4). Cyanophage composed 2% of total identified proteins at 100 m and 1% in the 105 m sediment trap sample (Fig. S3). These structural proteins could be indicative of detection of free viruses. However, we also detected proteins expected to be expressed only during the process of viral infection including a viral DNA maturase and a host-derived viral photosystem I P700 chlorophyll *a* protein (PsaB), both detected at 100 m [62, 63]. This is additional evidence for ongoing cyanophage infection in these waters. Viral lysis of cyanobacteria would inject organic matter into the ODZ. Our viral results are consistent with the hypothesis that in situ production by cyanobacteria in the ODZ leads to release of organic matter into the system.

### Impacts of in situ productivity on nitrogen cycling

Consistent with observations of active cyanobacterial oxygenic photosynthesis within the ODZ, combined STOX sensor depth profiles from five casts from BB2 indicate transitory nanomolar oxygen concentrations in the depth range of the secondary chlorophyll *a* maximum (Fig. 2) [54]. The creation of oxygen by photosynthesis may enable oxygen-requiring organisms to exist and thrive within the nominally anoxic ODZ. In particular, both ammonia and nitrite oxidization can occur at very low oxygen concentrations. The *K*_m_ for nitrite oxidizers at low oxygen is 0.5 ± 4.0 nM O_2_ (from two component fitting) and for ammonia oxidizers is 333 ± 130 nM [64]. Metagenomes indicate the aerobic chemoautotrophic nitrite oxidizer, *Nitrospina*, co-occurred with cyanobacteria in the ODZ (Fig. 2), and the aerobic ammonia-oxidizing Thaumarchaea were also found at lower abundances in the same depth range (Fig. 2) [34]. We also detected the nitrite-oxidizing protein nitrate oxidoreductase from *Nitrospina* at 100 m (Table S5). Thus, the presence of *Prochlorococcus* may contribute to nitrite oxidation in this ODZ. Measured potential oxidation rates from this same cruise are consistent with this idea [65]. Nitrite oxidation rates had their maximum of 120 nM day^−1^ at 105 m at BB2 and were undetectable at 150 m [65], below the LLIV/LLV *Prochlorococcus* maximum. Ammonia oxidation rates were 9 nM day^−1^ at 105 m (1/3 of their maximum) and were undetectable at 150 m, but were not measured in between [65]. Similarly, transcripts indicated activity of nitrite oxidizer *Nitrospina* and ammonia-oxidizing archaea at the secondary chlorophyll *a* maximum in 2014 [21]. Transcript evidence also supports the possibility of heterotrophic use of oxygen in anoxic waters at the secondary chlorophyll *a* maximum [21]. Our results are consistent with the idea that oxygen production from photosynthesis during the day by *Prochlorococcus* in the ODZ may fuel an oxygen-utilizing community under anoxic conditions, and thus the activity of bacteria and archaea at these depths may vary over a diel cycle.

Several lines of evidence indicate active production of N_2_ in the offshore region. We have previously reported the diverse community of denitrifiers identified at this station with metagenomics [34]. Here, in the metaproteomes at least six types of nitrate reductase proteins, including both types found in the ODZ SAR11 [50], were found in 145, 160, and 250 m samples (Table S5). Respiratory nitrate reductase proteins were also found in 105 m sediment trap material (Table S5). Anammox activity was also detected in the metaproteome, consistent with the previously described presence of *Candidatus* Scalindua in the water [34]. Proteins detected for the anammox process included hydrazine synthase (both subunits), hydroxylamine oxidoreductase, nitrite oxidoreductase, and an ammonium transporter, found at 145, 160, and 250 m in the water column (Table S5). Thus, the proteins detected here are consistent with in situ occurrence of both denitrification and anammox. Previously reported denitrification and anammox rates were 2.1 and 0.9 nM day^−1^, respectively, at 150 m at offshore station BB2 [5, 66]. Rates increased to 15.1 and 6.0 nM day^−1^, respectively, when excess sterile sediment trap material was added [5].

### Carbon flux and offshore production of N_2_

Despite low surface productivity and export, in the following calculation, we show that measured N_2_ gas concentrations and N_2_ production rates can be supported by the amount of organic C in the offshore ODZ without the need for transport of N_2_ from coastal waters. Since sediment trap organic C was 40% greater at 150 m than 105 m, in situ production may contribute to N_2_ production rates. We can estimate N_2_ production rates from organic matter fluxes using an equation for denitrification [67]:

$$\begin{array}{l}{\mathrm{C}}_{106}{\mathrm{H}}_{175}{\mathrm{O}}_{42}{\mathrm{N}}_{16}{\mathrm{P}} + 94.4\,{\mathrm{HNO}}_3 \to 106{\mathrm{CO}}_2 + 16{\mathrm{NH}}_3 + \\ {\mathrm{H}}_3{\mathrm{PO}}_4 + 109.2\,{\mathrm{H}}_2{\mathrm{O}} + 47.2{\mathrm{N}}_2,\end{array}$$

and assuming that anammox bacteria can use the ammonia produced in this organic matter degradation in a 1:1 ratio with nitrite to make N_2_. We use the difference between sinking C fluxes at 150 m (0.66 mmol C m^−2^ day^−1^) and 750 m (0.34 mmol C m^−2^ day^−1^), at the bottom of the ODZ, to estimate the amount of organic C consumed in the ODZ. However, sediment traps do not quantify all types of C fluxes. Both export of dissolved organic C through mixing and active C export by zooplankton migration could be important factors increasing the organic C flux [68]. Total organic C fluxes, including dissolved organics and active transport, are estimated to be 2–3 times larger than sediment trap fluxes in the North Pacific [68]. For our calculation, we double the amount of organic C consumed in the ODZ from sediment trap fluxes (0.32 mmol C m^−2^ day^−1^) to estimate total C fluxes (0.64 mmol C m^−2^ day^−1^). N_2_ production is much higher in the upper part of the ODZ compared to the lower [5]. If the majority of organic matter consumption occurred in the top 30% of the ODZ (200 m), our calculated N_2_ production rate in N units would be 4–6 nM N day^−1^, which is similar to measured rates (1.5–5 nM N day^−1^; [5]). The residence time of water in the ETNP ODZ in a 3-D model was 3.9 ± 0.8 years [69], allowing 6–9 μM N as N_2_ to build up in the top 200 m from consumption of organic C fluxes. Measured biological N_2_ was 10 μM N as N_2_ at 150 m at this station [34]. Organic matter fluxes like those seen here would allow offshore N_2_ production to create measured values of N_2_ gas. Thus, we have evidence from rate measurements and proteomics that N_2_ production occurs locally offshore and our calculations indicate that the majority of the N_2_ gas measured offshore could be produced in situ. The increase in sediment trap fluxes from 105 to 150 m suggests that in situ primary production accounted for 40% of these fluxes and thus of 40% of the N_2_ production. Given the extent of the secondary chlorophyll *a* maximum in the offshore ETNP ODZ (Fig. 1), the impact of in situ primary production in the ODZ could be significant. In corroboration, among three offshore stations examined in the ETSP, active anammox cells were only detected at the station with a deep cyanobacterial maximum within the ODZ [70]. Additionally, no N_2_ production was detected at stations in the offshore Southern ETNP where the top of the ODZ was at 340 m [71], a depth too deep for cyanobacteria to obtain light.

## Conclusions

We propose that offshore N loss processes were at least partly due to the influx of organic matter created by primary production inside the ODZ. Together, our metagenomic and metaproteomic data indicates that cyanobacteria were abundant in the upper ODZ and were actively photosynthesizing and dividing. According to metagenomic and metaproteomic data, cyanobacteria comprised 7% of prokaryotes and 17% of identified proteins on 100 m particles. Proteins derived specifically from this ODZ population were found in sediment traps, suggesting an in situ cyanobacterial contribution to particles. Detection of cyanophage proteins indicative of active infection and high corresponding cyanophage-to-cyanobacterial ratios suggest that cyanobacteria may have been experiencing viral-induced death. The release of organic matter from this process could have been a source of carbon fueling microbial activity within the ODZ. In support of this concept, the sediment trap-derived particle flux estimates at 150 m, right below the secondary chlorophyll maximum (100–140 m) were higher than those at 105 m, suggesting that cyanobacteria were actively contributing to particle flux there.

The thickness of the ETNP ODZ has increased over the past 40 years and the upper oxycline has become shallower [3]. Further shoaling of the ODZ into sunlit waters could increase the habitat of cyanobacteria and thus the amount of in situ primary production in the ODZ. The presence of significant primary production within the N-replete ODZ may contribute organic carbon used for N_2_ production, allowing N loss from oceanic ODZs to continue even with increasing N limitation in surface waters.

## Supplementary information

Supplemental Figures S1-S6

Table S1

Table S2

Table S3

Table S4

Table S5
