# Supplementary material for: Cyanobacteria and cyanophage contributions to carbon and nitrogen cycling in an oligotrophic oxygen-deficient zone
Source: ISME J. 2019 Jun 27;13(11):2714–26. doi: 10.1038/s41396-019-0452-6 (PMC6794308; doi:10.1038/s41396-019-0452-6)
Supplement: Supplementary file 1 — Supplemental Figures S1-S6 [file 41396_2019_452_MOESM1_ESM.docx]

Supplemental Figures for

**Cyanobacteria and cyanophage contributions to carbon and nitrogen cycling in an oligotrophic oxygen deficient zone**

Clara A. Fuchsman, Hilary I. Palevsky, Brittany Widner, Megan Duffy, Michael C.G. Carlson, Jacquelyn A. Neibauer**,** Margaret R. Mulholland, Richard G. Keil, Allan H. Devol, Gabrielle Rocap


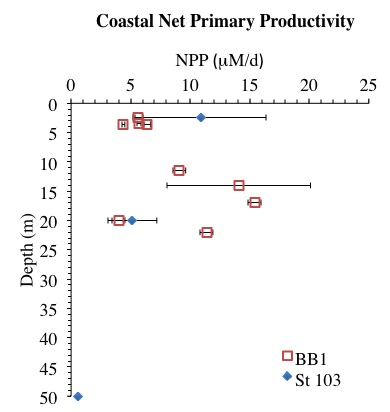


Figure S1. Coastal Net Primary Productivity as determined by ^13^C incubations.

Figure S2. Amino acid *psbD* maximum likelihood phylogenetic tree. ETNP indicates sequences assembled from metagenomic data.

Figure S3. Taxonomic identification of proteins with >1% coverage in meta-proteomic samples. Sample depth and type is listed at the bottom of the figure where Trap indicates sediment trap, 0.7 μm indicates GF/F filter and 0.2 μm indicates Sterivex filter.

Figure S4. Amino acid phylogenetic tree for DNA polymerase B for myoviruses. ETNP indicates sequences assembled from metagenomic data. Clade numbers are taken from Huang et al. (2015).

Figure S5. Amino acid phylogenetic tree for DNA polymerase A for podoviruses. ETNP indicates sequences assembled from metagenomic data.

Figure S6. Comparison of DNA polymerase phylotypes between particulate (>30 μm) and >0.2 μm water column samples. A.) 120m particles compared to anoxic waters (100m, 110m, and 120m) B) 120m particles compared to oxic waters above the ODZ (70m and 90m) and C) 100m particles compared to oxic waters above the ODZ. Each symbol represents the proportion of a phylotype of DNA polymerase. The black line represents the 1:1 line.
