# Supplementary material for: Cyanobacteria and cyanophage contributions to carbon and nitrogen cycling in an oligotrophic oxygen-deficient zone
Source: ISME J. 2019 Jun 27;13(11):2714–26. doi: 10.1038/s41396-019-0452-6 (PMC6794308; doi:10.1038/s41396-019-0452-6)
Supplement: Supplementary file 2 — Table S1 [file 41396_2019_452_MOESM2_ESM.docx]

Table S1. Station information and data for mixed layer gross primary production (GPP) and net community production (NCP) rates. Productivity rates integrate to the mixed layer depth (MLD). GPP is calculated from triple oxygen isotope (^17^∆) measurements, and also converted to rates equivalent to the ^13^C-NPP method. NCP (equivalent to carbon export from the mixed layer) is calculated from ΔO_2_/Ar (% biological oxygen supersaturation) measurements. ^2^

| Stn | Lati-tude | Longi-tude | Chl a (μg/ L)^1^ | MLD (m) | ^17^∆ (per meg) | GPP (mmol O_2_ m^-2^ d^-1^) | NPP (mmol C m^-2^ d^-1^) | ΔO_2_/Ar (%) | NCP (mmol C m^-2^ d^-1^) | Export efficiency (NCP/ NPP) |
| --- | --- | --- | --- | --- | --- | --- | --- | --- | --- | --- |
| 132^*^ | 18.8 | -104.1 | 20.27 | 11 | 149±32 |  | * | -8±2 | * | * |
| 133 | 18.4 | -105.0 | 1.07 | 14 | 59±7 | 182±29 | 67.3±10.9 | 2.7±0.002 | 16.1±3.3 | 0.24±0.03 |
| 134 | 18.1 | -105.4 | 0.27 | 30 | 39 | 71±11 | 26.3±4.0 | 0.3 | 1.2±1.6 | 0.04±0.06 |
| 135 | 17.6 | -106.0 | 0.14 | 34 | 28±6 | 41±7 | 15.0±2.4 | -0.3±0.4 | -1.3±1.5 | -0.08±0.10 |
| 136 | 17.0 | -106.5 | 0.13 | 36 | 38±5 | 65±9 | 24.2±3.5 | 0.1±0.4 | 0.3±1.5 | 0.01±0.06 |
| BB2 | 16.5 | -107.1 | 0.12 | 42 | 45 | 84±11 | 31.2±4.2 | 0.6 | 2.3±1.5 | 0.07±0.05 |
| 161 | 15.9 | -107.9 | 0.12 | 47 | 44±7 | 85±11 | 31.3±4.0 | 0.7±0.02 | 3.2±1.6 | 0.10±0.05 |
| 162 | 15.3 | -108.5 | 0.13 | 17 | 33±5 | 58±10 | 21.6±3.8 | 0.6±0.4 | 2.7±1.7 | 0.13±0.07 |
| 163 | 14.7 | -109.3 | 0.13 | 35 | 45±3 | 94±13 | 34.6±4.9 | 0.1±0.2 | 0.2±1.6 | 0.01±0.05 |
| 164 | 14.0 | -110.0 | 0.13 | 42 | 42±1 | 94±12 | 34.7±4.4 | 0.5±0.8 | 2.5±1.8 | 0.07±0.05 |

^1^Surface chlorophyll concentrations are from MODIS 8-day satellite data.

^2^Reported uncertainties in ^17^∆ and ΔO_2_/Ar are the standard deviation between replicates. Uncertainty in GPP, NPP, NCP and export efficiency represents uncertainty from all terms in the rate calculations, determined from a Monte Carlo analysis.

^*^NPP and NCP rates are not calculated for this most coastal station due to strong physical influence of upwelling on mixed layer ^17^∆ and ∆O_2_/Ar.
