# Supplementary material for: Cyanobacteria and cyanophage contributions to carbon and nitrogen cycling in an oligotrophic oxygen-deficient zone
Source: ISME J. 2019 Jun 27;13(11):2714–26. doi: 10.1038/s41396-019-0452-6 (PMC6794308; doi:10.1038/s41396-019-0452-6)
Supplement: Supplementary file 3 — Table S2 [file 41396_2019_452_MOESM3_ESM.docx]

Table S2. Station information and productivity measurements from ^13^C incubation analyses.

| Station | Latitude | Longitude | Mixed Layer (m) | Satellite chlorophyll a (μg/L) | Depth (m) | Productivity μM C d^-1^ |
| --- | --- | --- | --- | --- | --- | --- |
| St 103 | 19.8 | -105.8 | 18 | 12.9 | 2.5  20  50 | 11 ± 5.5  5 ± 2  0.5 ± 0.05 |
| BB1 | 20.1 | -106.0 | 13 | 13.3 | 2.5  3.5  11  14  17  20  22 | 5.6 ± 0.4  6.4±0.3, 5.7±0.2, 4.4±0.1  9.1 ± 0.5  14.1 ± 6.0  15.4 ± 0.6  4.0 ± 0.5  11.4 ± 0.5 |
| BB2 | 16.5 | -107.1 | 42 | 0.12 | 2  3  4  65  70 | 1.17 ± 0.04  1.1 ± 0.1  0.46 ± 0.02  1.26 ± 0.02, 0.9 ± 0.2  1.8 ± 0.2 |
| St 168 | 15.5 | -110.0 | 27 | 0.12 | 2  65 | 0.60 ± 0.05  0.67 ± 0.09 |
| St 173 | 18.0 | -110.0 | 18 | 0.14 | 2  50 | 1. ± 0.2   1.71 ± 0.08 |
