# Supplementary material for: Cyanobacteria and cyanophage contributions to carbon and nitrogen cycling in an oligotrophic oxygen-deficient zone
Source: ISME J. 2019 Jun 27;13(11):2714–26. doi: 10.1038/s41396-019-0452-6 (PMC6794308; doi:10.1038/s41396-019-0452-6)
Supplement: Supplementary file 4 — Table S3 [file 41396_2019_452_MOESM4_ESM.docx]

Table S3. Sample depth and filter pore sizes for metagenomic and metaproteomic samples.

|  | Metagenomes | | | Metaproteomes | | |
| --- | --- | --- | --- | --- | --- | --- |
| Station | 136 | BB2 | BB2 | BB2 | BB2 | BB2 |
| Size fraction | >0.2 μm | >30 μm | <30 μm - >0.2 μm | >0.7 μm | > 0.2 μm | Sediment trap |
| 55m |  |  |  | X |  |  |
| 60m | X |  |  |  |  |  |
| 70m | X |  |  |  |  |  |
| 90m | X |  |  |  |  |  |
| 100m | X | X |  | X | X |  |
| 105m |  |  |  |  |  | X |
| 110m | X |  |  |  |  |  |
| 120m | X | X | X |  |  |  |
| 140m | X |  |  |  |  |  |
| 145m |  |  |  | X |  |  |
| 150m |  | X |  |  | X |  |
| 160m | X |  |  | X |  |  |
| 250m |  |  |  | X |  |  |
| 750m |  |  |  |  |  | X |
